# Cav1.2 facilitates the binding and internalisation of porcine epidemic diarrhoea virus

**DOI:** 10.1186/s13567-025-01615-8

**Published:** 2025-09-25

**Authors:** Xinxin Wang, Guilan Guo, Shutong He, Kaili Wang, Jianing Chen, Guijie Guo

**Affiliations:** 1 Key Laboratory of Animal Pathogen Infection and Immunology of Fujian Province, College of Animal Sciences, Fujian Agriculture and Forestry University, Fuzhou, China; 2 Fujian Province Joint Laboratory of Animal Pathogen Prevention and Control of the ‘Belt and Road’, College of Animal Sciences, Fujian Agriculture and Forestry University, Fuzhou, China; 3 Key Laboratory of Fujian-Taiwan Animal Pathogen Biology, College of Animal Sciences, Fujian Agriculture and Forestry University, Fuzhou, China; 4 State Key Laboratory for Animal Disease Control and Prevention, College of Veterinary Medicine, Lanzhou University, Lanzhou, China; 5 Lanzhou Veterinary Research Institute, Chinese Academy of Agricultural Sciences, Lanzhou, China; 6 Xinxiang Key Laboratory of Inflammation and Immunology, School of Medical Technology, Henan Medical University, Xinxiang, China

**Keywords:** Cav1.2, PEDV, entry factor, viral binding, internalisation

## Abstract

Porcine epidemic diarrhoea virus (PEDV) has caused substantial economic losses to the global swine industry. The PEDV spike (S) protein mediates viral entry by interacting with host receptors. However, the molecular mechanisms underlying PEDV entry remain incompletely understood. In this study, we identified the L-type calcium channel Cav1.2 as a critical host factor for PEDV entry. Depletion of Cav1.2 significantly suppressed PEDV replication. Additionally, treatment with the FDA-approved Cav1.2 blocker diltiazem inhibited PEDV replication and disrupted early infection events. Mechanistically, we found that Cav1.2 interacts with the PEDV S1 subunit. Both Cav1.2 knockdown and diltiazem treatment substantially reduced the binding and internalisation of PEDV. These findings reveal that Cav1.2 is a key host factor for PEDV binding and internalisation via interaction with the viral S protein, and suggest that Cav1.2 may serve as a promising target for antiviral drug development against PEDV.

## Introduction

Porcine epidemic diarrhoea (PED) is an acute and highly contagious intestinal disease characterised by diarrhoea, vomiting, dehydration and high mortality in suckling piglets [1–4]. This condition has resulted in substantial economic losses within the global swine industry [5, 6]. The causative agent of PED is the porcine epidemic diarrhoea virus (PEDV), which is usually transmitted through the faecal-oral route [7–10]. PEDV is an enveloped, single-stranded, positive-sense RNA virus belonging to the genus *Alphacoronavirus*, which encodes four major structural proteins: the spike protein (S), membrane protein (M), envelope protein (E), and nucleocapsid protein (N) [10, 11]. Among these, the S protein is essential for viral entry into host cells and is the primary target of neutralising antibodies [2, 12, 13]. Studies have shown that different regions of the S protein contain multiple epitopes for neutralising antibodies, which may exhibit antigenic differences among various PEDV strains [14–16].

The first step in viral infection is entry into target cells, initiated by the binding and interaction of viral envelope glycoproteins with cellular receptors [17, 18]. The PEDV S protein is the principal mediator of virus entry into the target cells. The N-terminal S1 subunit is responsible for receptor binding, while the C-terminal membrane-anchored S2 domain facilitates membrane fusion [1, 11]. Entry receptors typically determine viral host range or species-specific tropism. Porcine aminopeptidase N (APN) was initially identified as a receptor for PEDV [19–21]. However, a recent study demonstrated that APN is not a functional receptor for PEDV, but instead promotes PEDV infection by mediating internalisation in susceptible cells [22]. Other host factors are also involved in the PEDV invasion process [23–32]. Despite existing reports on PEDV entry factors, the underlying entry mechanism remains a matter requiring further study. Elucidating the mechanisms of receptor recognition, cell binding, and internalisation is essential for developing novel entry inhibitors and vaccines.

The L-type calcium channel Cav1.2 pore-forming subunit (Cav1.2) is a voltage-dependent calcium channel that plays a crucial role in viral infection [33–35]. Cav1.2 is a typical cytomembrane-bound protein encoded by the calcium voltage-gated channel subunit alpha 1 C gene [36]. Studies have shown that Cav1.2 acts as an entry receptor for the influenza virus; its hemagglutinin (HA) can bind to Cav1.2, triggering intracellular calcium oscillations that facilitate viral entry and replication [34, 37]. Cav1.2 also plays a key role in SARS-CoV-2 infection, interacting with the viral spike protein and affecting the attachment and internalisation of SARS-CoV-2 [35]. However, its role in PEDV infection remains undefined.

In this study, we identified Cav1.2 as essential for PEDV infection, and confocal microscopy data showed that Cav1.2 affects PEDV binding. Furthermore, the FDA-approved drug diltiazem, a Cav1.2 blocker, reduced both PEDV binding and cell entry. Our data indicate that Cav1.2 functions as an entry receptor of PEDV. Together, these findings enhance the current understanding of the PEDV entry process and may support the development of targeted entry inhibitors for PEDV infection.

## Materials and methods

### Cells, viruses, and plasmids

Vero-E6 and HEK293T cells were maintained in Dulbecco’s modified Eagle’s medium (DMEM) supplemented with 10% foetal bovine serum (FBS), 1% penicillin/streptomycin, And L-glutamine, in 5% CO_2_. The PEDV CV777 strain was kindly provided by Jianing Chen (State Key Laboratory for Animal Disease Control and Prevention, College of Veterinary Medicine, Lanzhou University; Lanzhou Veterinary Research Institute, Chinese Academy of Agricultural Sciences, Lanzhou, China). The propagation of PEDV was performed using Vero-E6 cells. Progeny virus titres were determined by the Reed–Muench method. Cav1.2 cDNA and the PEDV S1 subunit (aa 26–734) were cloned into pCAGGS-Flag or pCAGGS-Myc vectors, as indicated, and confirmed by sequencing analysis.

### Virus infection assay

For PEDV infection, cells were infected at the indicated time and multiplicity of infection (MOI) for 1 h at 37 °C. The cells were then washed three times with phosphate-buffered saline (PBS), And medium containing 2% FBS was added. At 24 h post-infection, the infected cells were harvested, and viral RNA levels and PEDV nucleocapsid protein (NP) expression were measured using quantitative polymerase chain reaction (qPCR) and western blotting assays, respectively. Viral titres were determined using the Reed–Muench method.

### Viral growth curve of PEDV

Vero-E6 cells were seeded into 24-well plates for 16 h, after which the medium was removed and cells were preincubated with vehicle or diltiazem (150 μΜ) for 1 h at 37 ℃. Vero-E6 cells were then infected with PEDV (MOI = 5) for 1 h at 37 ℃, with diltiazem present throughout the infection period. Cells And supernatants were harvested at 1 h, 6 h, 12 h, And 24 h post-infection. Viral RNA levels in the cell lysates, relative to glyceraldehyde-3-phosphate dehydrogenase (GAPDH), were measured by qPCR. Viral titres were determined using the Reed–Muench method.

### RNAi assay

siRNA transfections were performed in 24-well plates using Lipofectamine RNAiMAX transfection reagent (Thermo Fisher Scientific), according to the manufacturer’s instructions. Briefly, siRNA (Sigma) targeting Cav1.2 or non-targeting siRNA was mixed with OptiMEM medium (Invitrogen) containing Lipofectamine RNAiMAX transfection reagent on 24-well plates. After 30 min of incubation at room temperature, cells were seeded into the siRNA-coated 24-well plates. At 24 h, Cav1.2 mRNA expression was assessed using qPCR. At 60 h post-transfection, cells were infected with PEDV (MOI = 0.01) for further studies. The siRNA sequences were as follows: siCav1.2, Sense: 5'-CCUACUUCGUGUCCCUCUUdTdT-3', Antisense: 5'-AAGAGGGACACGAAGUAGGdTdT-3'.

### Inhibitor assay

Vero-E6 cells were preincubated with vehicle or diltiazem (Sigma, D2521; 150 μΜ) for 1 h at 37 ℃, after which the virus infection assay was performed. Vero-E6 cells were infected with PEDV for 1 h at 37 ℃. Diltiazem was present throughout the infection period. At 24 h post-infection, the supernatants and cells were harvested to measure viral titres, viral RNA levels, and PEDV NP protein expression, respectively.

### Time-of-addition assay

Vero-E6 cells were infected with PEDV (MOI = 5) for 1 h at 37 ℃. After washing, diltiazem (150 μM) was added at -1 h, 1 h, 2 h, or 4 h post-infection. The virus in the cell lysate was detected by qPCR at 6 h post-infection.

### Viral binding assay

Vero-E6 cells were seeded into 24-well plates And either transfected with the indicated siRNA for 60 h or pretreated with diltiazem for 1 h. Cells were placed on ice for 20 min, the medium was removed, And 200 μL of PEDV (MOI = 0.01, 1, 10, or 50) was added at 4 °C for 1 h. After three washes with pre-chilled PBS to remove the unbound virions, cells and bound virions were harvested for qPCR, reverse transcription polymerase chain reaction (RT-PCR), and western blotting assays.

### Viral entry assay

Vero-E6 cells were seeded into 24-well plates And placed on ice for 20 min. The media were removed from the wells, And 200 μL of PEDV (MOI = 10) was added to the cells at 4 ℃ for 1 h. After unbound virions were removed by extensive washing with chilled PBS, cells in group A were incubated in medium containing diltiazem at 37 ℃ for 5 h to allow for the internalisation of bound virus. In group B, cells were first transferred to 37 ℃ for 1 h to culture, after which diltiazem was added And the culture continued for An additional 4 h. The harvested cells were analysed by qPCR and western blotting assays.

### Immunofluorescence staining and confocal microscope

Vero-E6 cells were cultured in 35 mm confocal petri dishes or tissue culture plates. Following viral binding assays, cells were immediately fixed with 3% paraformaldehyde for 15 min at room temperature, permeabilised with 0.1% Triton X-100 in PBS for 10 min, And incubated with 1% BSA for 30 min to block non-specific Antibody binding. Cells were then incubated overnight at 4 °C with anti-PEDV NP mouse polyclonal antibody (Zoonogen, M100047), washed And stained with Alexa Fluor 488 goat anti-mouse (Thermo Fisher Scientific, A-11001) for 1 h. The plasma membrane was stained with Dil (Beyotime, C1991S), and nuclei were visualised by staining with DAPI. Fluorescence intensity was quantified using a Leica STELLARIS8 FALCON microscope. Signal intensity of cell-bound PEDV was measured in at least 100 cells per sample using LAS X Office software.

Vero-E6 cells were transfected with Cav1.2-Flag and PEDV S1-Myc plasmids for 24 h, then fixed And permeabilised with 0.1% Triton X-100, followed by incubation with anti-Flag (Sigma, F1804) or anti-Myc (Proteintech, 16286–1-AP) primary Antibodies overnight at 4 ℃. After washing, cells were incubated with secondary antibodies (Thermo Fisher Scientific, A-11001, A-11012) at room temperature for 1 h. Nuclei were visualised by staining with DAPI.

### Real-time quantitative PCR

To detect Cav1.2 mRNA and viral RNA levels, total RNA was isolated from cells using TRIzol reagent. Two micrograms of total RNA were used for reverse transcription (Vazyme, R123).

Relative mRNA expression was analysed using SYBR Green qPCR Master Mix (Vazyme, Q711) with the Cav1.2 and PEDV NP primers.

The 2^−∆∆CT^ method was used to calculate the relative gene expression level, with GAPDH as the internal control. The qPCR primers used were as follows: Cav1.2: forward (5'-CGAGGAAGAGGAGAAGGAGAGAA-3') and reverse (5'-TCAGCCGTGATGGATTTCAG-3'). GAPDH: forward (5'-TCAACTACATGGTTTACATGTTCCA-3') and reverse (5'-CAAACATAGGGGCGTCAGCA-3'); and PEDV NP: forward (5'-GCTATGCTCAGATCGCCAGT-3') and reverse (5'-TCTCGTAAGAGTCCGCTAGCTC-3').

### Co-immunoprecipitation assay

HEK293T cells were seeded into 6-well plates and transfected with plasmids using Lipo8000 transfection reagent (Beyotime, C0533) according to the manufacturer’s instructions. At 48 h post-transfection, cells were lysed in NP-40 lysis buffer for 1 h at 4 ℃. The supernatant was collected And mixed with 40 μL of protein G agarose (Abmart, A10001) for 4 h at 4 °C to remove non-specific binding proteins. After washing, the supernatant was mixed with anti-Flag antibody-conjugated agarose beads (Biolinkedin, L-1109) for 6 h at 4 ℃. Beads were isolated by centrifugation, washed five times with lysis buffer, and used for sodium dodecyl sulfate–polyacrylamide gel electrophoresis (SDS–PAGE) and western blotting.

### Western blotting

Cell lysate was diluted in denaturing SDS gel loading buffer And boiled for 15 min. After denaturing, samples were loaded onto an 8% gel (Yeasen, 20324ES62) for SDS–PAGE and separated by electrophoresis. Proteins were transferred to a polyvinylidene difluoride (PVDF) membrane (Merck Millipore, ISEQ00010). The PVDF membrane was blocked in 5% skim milk in PBS containing 0.1% Tween 20, then incubated with the following primary antibodies: anti-Flag (GenScript, A00187), anti-Myc (GenScript, A00172), anti-PEDV NP (Zoonogen, M100047), and anti-GAPDH (Servicebio, GB15002-100). Membranes were then washed three times with PBS and incubated with horseradish peroxidase (HRP)-conjugated goat anti-mouse antibody (GenScript, A00160) and goat anti-rabbit antibody (GenScript, A00098). After three washes with PBS containing 0.1% Tween 20 (PBST), target protein bands were detected by using an enhanced chemiluminescence (ECL) reagent.

### Statistical analysis

Quantitative data are presented as means ± standard deviations (SD) from three independent experiments or replicates. Statistical analysis of normalised data was performed in Microsoft Excel using an unpaired two-tailed Student’s *t*-test. The statistical details are provided in the figure legends. Significance levels were defined as follows: ns, not significant, p* < 0.05, p** < 0.01, p*** < 0.001.

## Results

### Cav1.2 is required for PEDV infection

**Figure 1:**
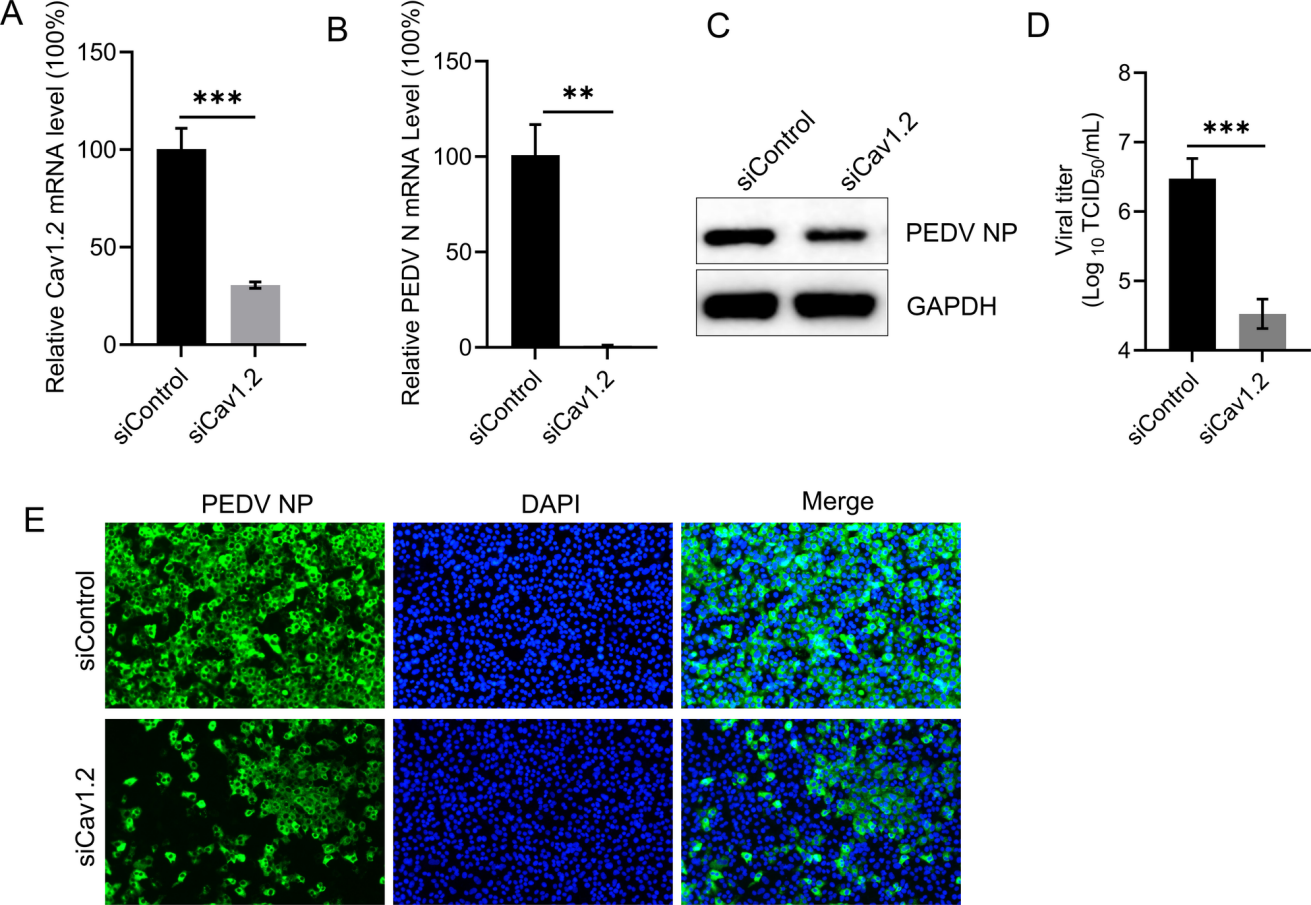
**Silencing Cav1.2 expression inhibits PEDV infection.** **A** The Cav1.2 mRNA level in the indicated siRNA-transfected Vero-E6 cells was measured by qPCR. siCav1.2, siRNAs specific for Cav1.2 mRNA; siControl, scrambled RNA. **B**–**D** Cav1.2-silenced Vero-E6 cells were infected with PEDV (MOI = 0.01) for 24 h, then the viral RNA (**B**), PEDV NP protein levels in the cells (**C**), and the viral titres of the supernatants (**D**) were determined by qPCR, western blotting and TCID_50_ assays, respectively. **E** Cav1.2-silenced Vero-E6 cells were infected with PEDV for 24 h at 37 °C. The cell nuclei were stained (blue), and the infected cells were stained with a PEDV NP antibody (green), and representative images are shown. The data shown are the means ± SDs of three independent experiments or replicates. The two-tailed unpaired Student’s *t*-test was used for the statistical analysis. p** < 0.01, p*** < 0.001

To determine whether Cav1.2 plays a role in PEDV infection, we knocked down its expression by transfecting Vero-E6 cells using siRNA targeting Cav1.2 or a non-targeting control. Real-time quantitative PCR (qPCR) analysis confirmed that Cav1.2 mRNA expression was significantly reduced in Vero-E6 cells at 24 h post-transfection (Figure 1A). At 60 h post-transfection, cells were infected with PEDV (MOI = 0.01). Viral replication was assessed by qPCR, western blotting, TCID_50_ assay, and microscopy analysis. Knockdown of Cav1.2 markedly inhibited PEDV replication in the cells (Figures 1B And 1C). Infectious titres in supernatants of the infected cells were measured by TCID_50_ assay at 24 h post-infection. Compared with siControl-transfected cells, Cav1.2 depletion significantly reduced the viral titres (Figure 1D). Additionally, microscopy Analysis at 24 h post-infection showed that Cav1.2 knockdown significantly reduced PEDV NP protein levels in infected Vero-E6 cells (Figure 1E). These findings indicate that Cav1.2 is required for efficient PEDV infection.

### Diltiazem inhibits PEDV infection

**Figure 2:**
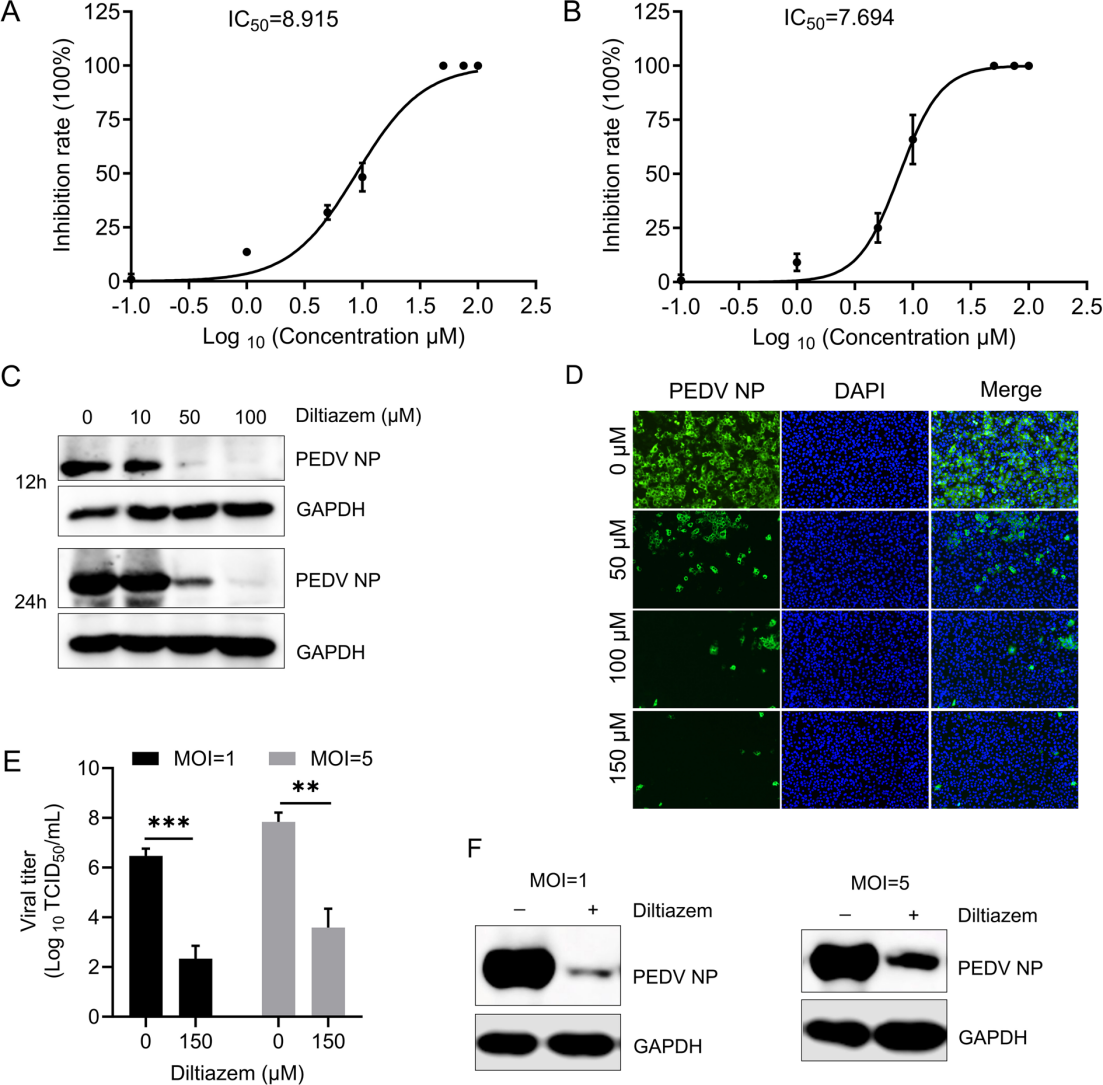
**Diltiazem inhibits PEDV infection in Vero-E6 cells.** **A** and **B** IC_50_ of diltiazem against PEDV infectivity. Vero-E6 cells were treated with vehicle or diltiazem at the indicated concentrations for 1 h, and then infected with PEDV (MOI = 0.01). The cells And supernatants were harvested at 24 h post-infection to determine viral RNA levels (**A**) and virus titres (**B**). IC_50_: 50% inhibitory concentration. **C** Vero-E6 cells were treated with diltiazem for 1 h, And then infected with PEDV at An MOI of 0.01. Cells were harvested at 12 h And 24 h post-infection for western blotting analysis. **D** Vero-E6 cells were infected with PEDV for 24 h, then the cell nuclei were stained (blue), and the infected cells were stained with a PEDV NP antibody (green), and representative images are shown. **E** and **F** Vero-E6 cells were treated with diltiazem for 1 h, And then infected with PEDV at An MOI of 1 or 5. The supernatants And cells were harvested at 24 h post-infection for TCID_50_ (**E**) and western blotting (**F**) assays, respectively. The data shown are the means ± SDs of three independent experiments or replicates. The two-tailed unpaired Student’s *t*-test was used for the statistical analysis. p** < 0.01, p*** < 0.001

To further evaluate the role of Cav1.2 in PEDV infection, we performed an inhibitor assay in Vero-E6 cells using the Cav1.2 inhibitor diltiazem. Diltiazem is a blocker of the L-type calcium channel Cav1.2 pore-forming subunit [38, 39]. Vero-E6 cells were treated with diltiazem at various concentrations for 1 h at 37 ℃, then infected with PEDV (MOI = 0.01). Infectious titres in the supernatants of the infected cells were evaluated at 24 h post-infection. Our previous study showed that the 50% cytotoxic concentration (CC_50_) of diltiazem in Vero-E6 cells was 279.2 μΜ [35]. The 50% maximal inhibitory concentration (IC_50_) of diltiazem for PEDV infection was 8.915 μM (based on viral RNA levels) And 7.694 μM (based on the virus titres) (Figures 2A And 2B). The inhibitory effect of diltiazem was further assessed by western blotting and microscopy analysis. The results showed that diltiazem significantly inhibited PEDV infection in a dose-dependent manner (Figures 2C And 2D). In addition, Vero-E6 cells pretreated with diltiazem were infected with PEDV at An MOI of 1 or 5, and diltiazem continued to inhibit infection at higher infection doses (Figures 2E And 2F). The results further support that diltiazem significantly inhibits PEDV infection.

### Diltiazem affects the early stage of PEDV infection

**Figure 3:**
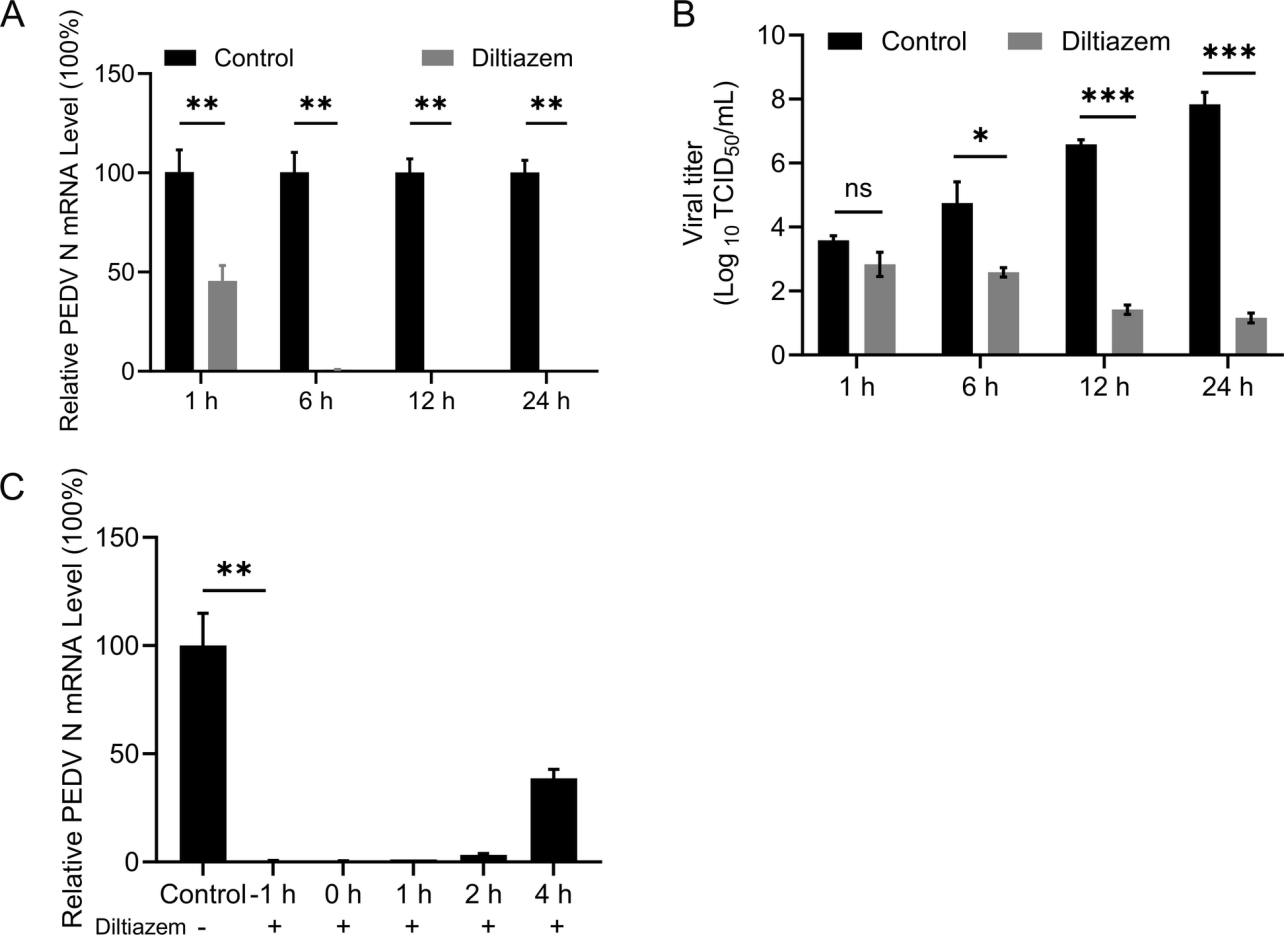
**Diltiazem inhibits the early stage of PEDV infection.** **A** and **B** Vero-E6 cells were incubated with diltiazem for 1 h, And then infected with PEDV at An MOI of 5. At the indicated time points post-infection, the viral RNA levels in the cell lysate (**A**) and the viral titres in the supernatants (**B**) were determined by qPCR and TCID_50_ assays. **C** Vero-E6 cells were infected with PEDV (MOI = 5), and diltiazem was added at −1 h, 0 h, 1 h, 2 h, or 4 h post-infection. The viral RNA level in the cell lysate was determined at 6 h post-infection by qPCR. The data shown are the means ± SDs of three independent experiments or replicates. The two-tailed unpaired Student’s *t*-test was used for the statistical analysis. ns, not significant, p* < 0.05, p** < 0.01, p*** < 0.001

We next investigated the specific stage at which Cav1.2 regulated PEDV infection. Vero-E6 cells were treated with 150 μΜ diltiazem for 1 h, then infected with PEDV (MOI = 5). Viral RNA level in the cell lysate and infectious titres in supernatants were determined by qPCR and TCID_50_ assays at 1 h, 6 h, 12 h, And 24 h post-infection, respectively. Compared with control cells (0 μM), diltiazem-treated cells showed significantly reduced viral RNA level as early as 1 h post-infection (Figure 3A), And viral titres were significantly lower at 6 h post-infection (Figure 3B), indicating that diltiazem inhibits the early stage of PEDV infection.

We further performed a time-of-addition assay to test whether diltiazem inhibits an early step of PEDV infection. Cells were treated with diltiazem at -1 h, 0 h, 1 h, 2 h, And 4 h after PEDV infection (MOI = 5). Viral RNA levels in cell lysates were assessed by qPCR at 6 h post-infection. We found that compared with the viral RNA level in control cells, the addition of diltiazem at -1 h, 0 h, 1 h, 2 h, or 4 h post-infection significantly decreased viral RNA levels (Figure 3C). These results indicate that diltiazem acts at an early stage of PEDV infection, suggesting that Cav1.2 may be involved in this process.

### Cav1.2 interacts with PEDV S protein

**Figure 4:**
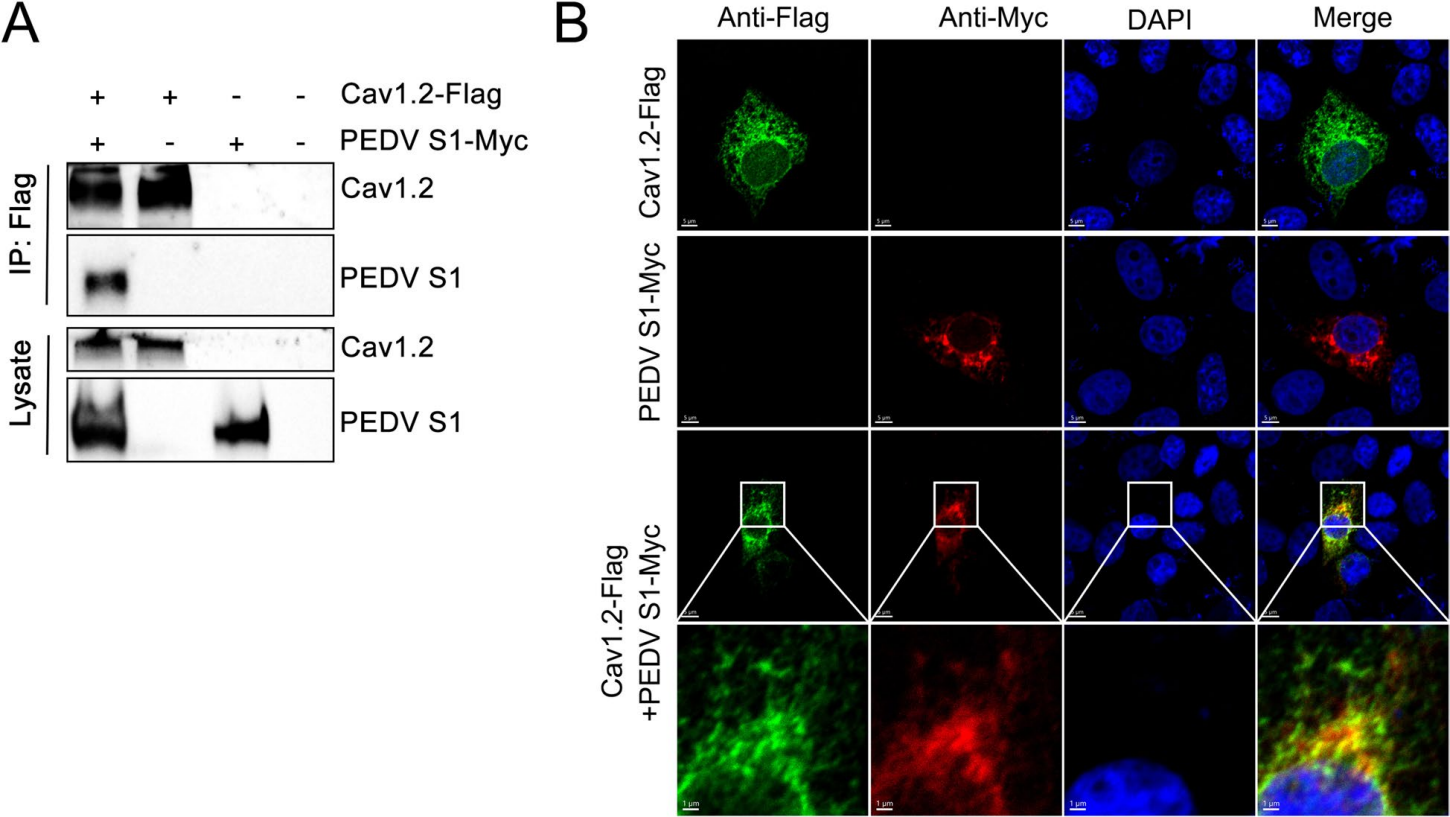
**Cav1.2 interacts with PEDV S1 protein.** **A** HEK293T cells were co-transfected with Cav1.2-Flag and PEDV S1-Myc plasmids and then subjected to co-immunoprecipitation assays using anti-Flag agarose beads. Representative western blotting of whole-cell lysates and eluates after co-immunoprecipitation are shown. **B** Vero-E6 cells were transfected with Cav1.2 and PEDV S1 plasmids, fixed And subjected to incubation with the indicated primary Antibody and corresponding secondary antibody, followed by visualisation by confocal immunofluorescence microscopy. Scale bar, 5 µm

The coronavirus spike (S) protein serves as the primary mediator of viral entry, facilitating both receptor recognition and membrane fusion [40]. The PEDV S protein is structurally organised into functional S1 and S2 subdomains [41]. The S1 subunit is responsible for host cell attachment, while S2 mediates membrane fusion [42]. Based on these findings, we hypothesised that Cav1.2 may function as an entry factor for PEDV by interacting with the S1 subunit. To test this hypothesis, we performed co-immunoprecipitation assays. HEK293T cells were co-transfected with Flag-tagged Cav1.2 (Cav1.2-Flag) and Myc-tagged PEDV S1 (S1-Myc), followed by immunoprecipitation (IP) assays and western blotting. As shown in Figure 4A, IP of Flag-Cav1.2 effectively pulled down PEDV S1, indicating an interaction between Cav1.2 and the PEDV S1 protein. Additionally, we performed confocal microscopy assays to examine the colocalisation of Cav1.2 and PEDV S1 proteins. Vero-E6 cells were transfected with Cav1.2-Flag and PEDV S1-Myc plasmids and subjected to confocal immunofluorescence assay. Cav1.2 was observed to colocalise with PEDV S1 protein (Figure 4B). These results suggest that Cav1.2 may contribute to PEDV entry via infection with the viral S protein.

### Diltiazem inhibits PEDV binding

**Figure 5:**
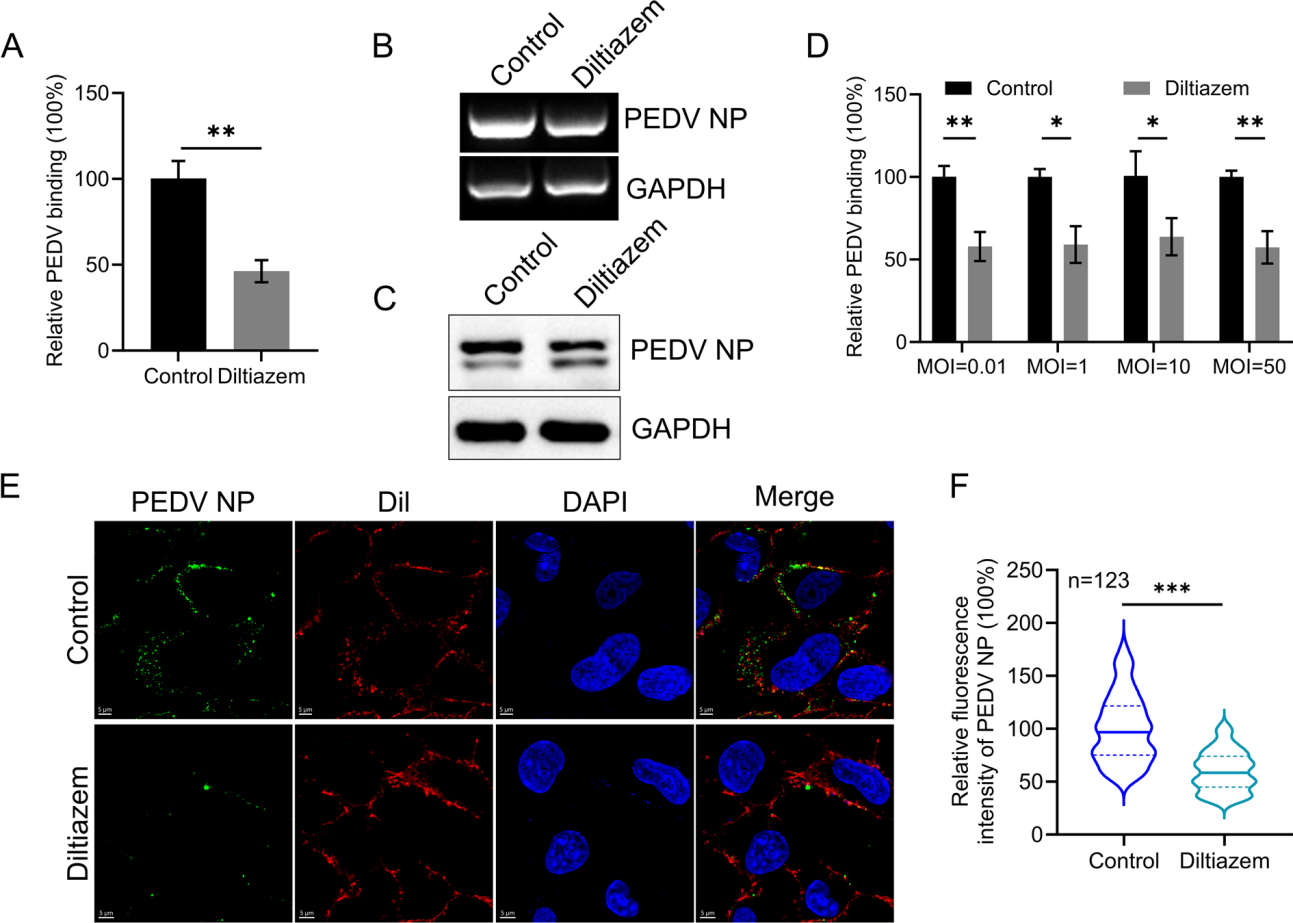
**Diltiazem inhibits PEDV binding.** **A**–**C** Diltiazem-preincubated Vero-E6 cells were infected with PEDV (MOI = 5) at 4 ℃ for 1 h. The viral RNA levels and PEDV NP protein expression were measured by qPCR (**A**), RT-PCR (**B**), and western blotting (**C**) assays, respectively. **D** Diltiazem preincubated Vero-E6 cells were infected with PEDV at An MOI of 50, 10, 1, or 0.01. The viral RNA level in the cell lysate was measured by qPCR. **E** Vero-E6 cells were treated and infected as described in (**A**). The cells were incubated with An Anti-PEDV NP mouse antibody, and visualised with Alexa Fluor 488-conjugated goat anti-mouse IgG (green). The plasma membrane was stained with Dil (Red). Cell nuclei were stained with DAPI. Representative images are shown. **F** The fluorescence intensities of cell-bound PEDV in 123 cells per sample were quantified. The two-tailed unpaired Student’s *t*-test was used for the statistical analysis. p* < 0.05, p** < 0.01, p*** < 0.001

The initial step in PEDV infection is binding to the host cell surface via the S protein, followed by viral entry and replication [11, 12]. To determine whether diltiazem inhibits PEDV binding, we performed viral binding assays. Diltiazem-treated cells were incubated with PEDV (MOI = 5) at 4 ℃ for 1 h, then immediately washed with chilled PBS to remove any unbound viruses. The viral RNA level in the cell lysate was determined using qPCR. We found that, compared to control cells, the viral RNA level in diltiazem-treated cells was significantly decreased, indicating that diltiazem inhibits PEDV binding (Figure 5A). In addition, we performed RT-PCR and western blotting assays to evaluate the cell surface-bound virus. Diltiazem-treated cells showed a significant reduction in both viral RNA levels and PEDV NP protein expression compared with control cells (Figures 5B And 5C).

We further performed viral binding assays using PEDV at MOIs of 0.01, 1, 10, or 50 in Vero-E6 cells as described above. Compared with control cells, diltiazem-treated cells showed significantly reduced viral RNA levels at all tested MOIs in Vero-E6 cells, indicating that the inhibition of PEDV binding by diltiazem is independent of the viral infection dose (Figure 5D). Furthermore, we performed a microscopy-based assay to observe the PEDV viral particles on the cell surface. Control and diltiazem-treated cells were infected with PEDV, then fixed and subjected to immunofluorescence assays using an antibody against the PEDV NP to visualise the viral particles. The plasma membrane was stained with Dil, and fluorescence intensity was quantified for each cell. PEDV fluorescence intensity was significantly lower in diltiazem-treated cells than in control cells (Figures 5E And 5F). These data further confirm that diltiazem inhibits PEDV binding.

### Knockdown of Cav1.2 inhibits the binding of PEDV

**Figure 6:**
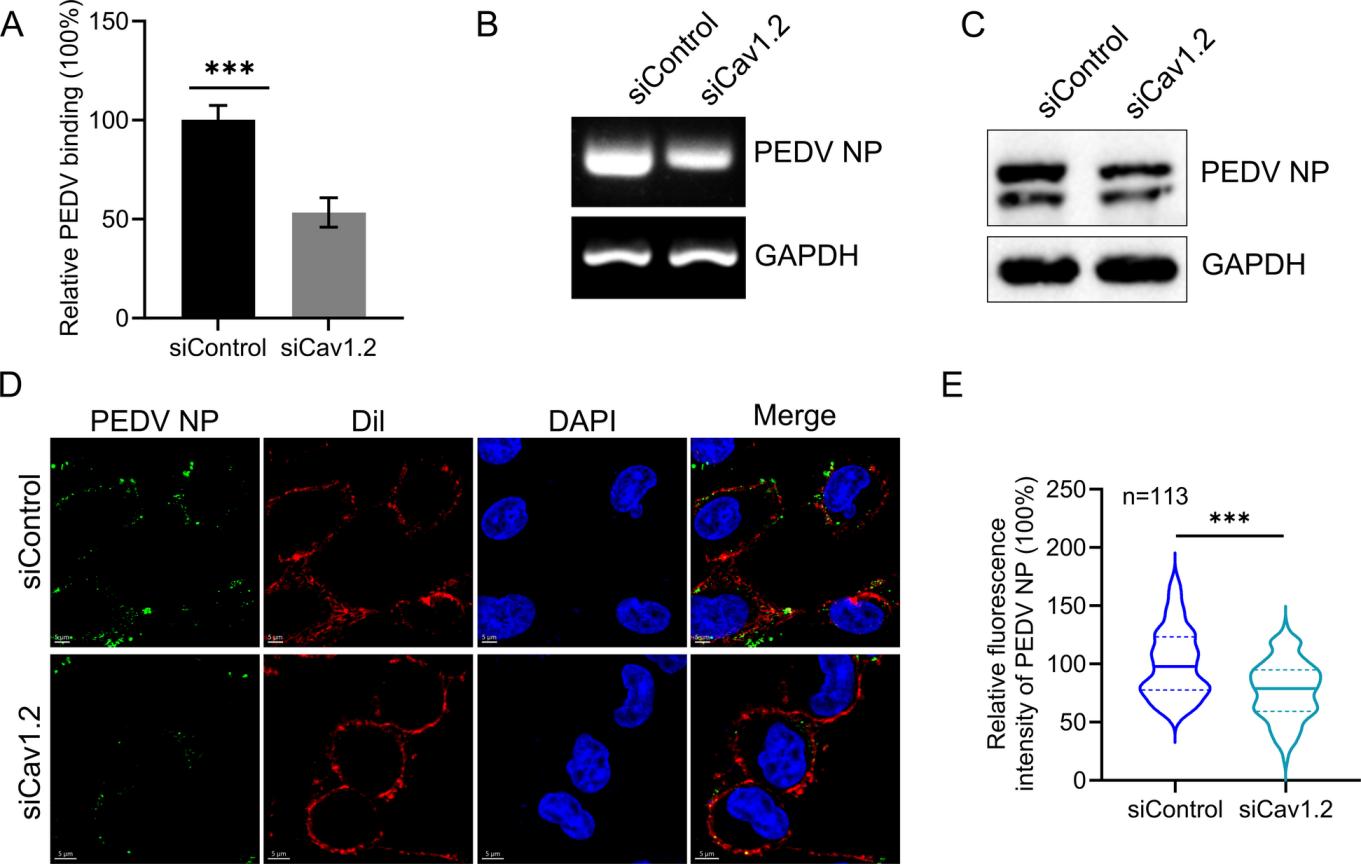
**Knockdown of Cav1.2 inhibits the binding of PEDV.** **A**–**C** Cav1.2-silenced cells were incubated with PEDV (MOI = 5) for 1 h at 4 ℃. The viral RNA levels and PEDV NP protein expression were detected by qPCR (**A**), RT-PCR (**B**), and western blotting (**C**) assays, respectively. **D** Vero-E6 cells were treated and infected as described in (**A**). The cells were incubated with An Anti-PEDV NP mouse antibody, and visualised with Alexa Fluor 488-conjugated goat anti-mouse IgG (green). The plasma membrane was stained with Dil (Red). Cell nuclei were stained with DAPI. Representative images are shown. **E** The fluorescence intensities of cell-bound PEDV in 113 cells per sample were quantified. The two-tailed unpaired Student’s *t*-test was used for the statistical analysis. p*** < 0.001

To further confirm the role of Cav1.2 in PEDV infection, we performed viral binding assays in siCav1.2-transfected cells. Vero-E6 cells were transfected with siRNA targeting Cav1.2 or control, and then subjected to a viral binding assay. Compared with siControl-transfected cells, siCav1.2-transfected cells showed significant downregulation of both the viral RNA levels and PEDV NP protein expression (Figures 6A–C). In addition, microscopy assay analysis revealed that the fluorescence intensity of PEDV was significantly lower in Cav1.2-silenced cells compared to control cells (Figures 6D And 6E). These data suggest that Cav1.2 depletion suppresses PEDV binding.

### Cav1.2 participates in the entry of PEDV

**Figure 7:**
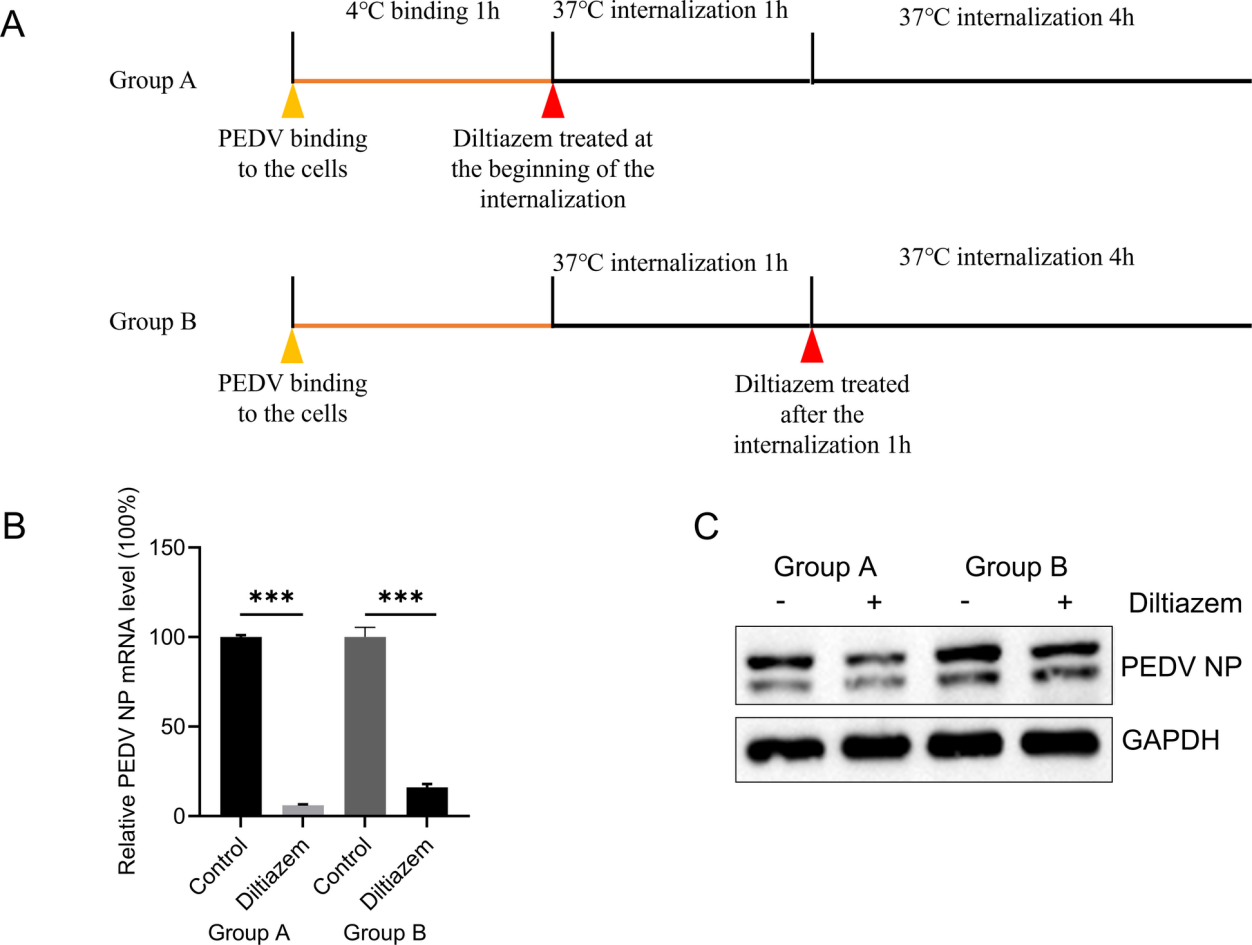
**The role of Cav1.2 in PEDV internalisation.** **A** Schematic of diltiazem treatment of PEDV-infected Vero-E6 cells. **B** and **C** Vero-E6 cells were incubated with PEDV (MOI = 5) for 1 h at 4 ℃, then washed with PBS to remove unbound virus. The cells were then subjected to one of two treatments: diltiazem added at the beginning of internalisation (group A), or 1 h after internalisation began (group B). They were incubated at 37 °C and finally collected for testing by qPCR (**B**) and western blotting (**C**) assays. The two-tailed unpaired Student’s *t-*test was used for the statistical analysis. p*** < 0.001

We next investigated the role of Cav1.2 in the entry of PEDV. An experiment was designed to evaluate the effect of Cav1.2 inhibition on viral internalisation. After incubation with PEDV at 4 ℃ for 1 h, group A cells were treated with diltiazem And transferred to 37 ℃ for a further 5 h of culture. In group B, cells were first incubated at 37 ℃ for 1 h, after which diltiazem was added, And the culture was continued for An additional 4 h (Figure 7A). As shown in Figures 7B And 7C, both treatment groups showed significant suppression of PEDV RNA and NP protein levels. Notably, group A exhibited a more pronounced inhibitory effect than group B, suggesting a critical role of Cav1.2 during viral internalisation. These results indicate that inhibition of Cav1.2 activity impairs PEDV internalisation, thereby reducing the efficiency of virus infection.

## Discussion

In this study, we found that knockdown of Cav1.2 significantly suppressed PEDV replication and that Cav1.2 interacts with the PEDV S1 subunit. Diltiazem, an FDA-approved blocker of Cav1.2, reduced PEDV infection in cells and inhibited cell binding and internalisation of PEDV.

Cav1.2 knockdown produced similar effects to diltiazem treatment with respect to PEDV binding and internalisation. These findings identify the L-type calcium channel Cav1.2 as a novel host entry receptor for PEDV, expanding our understanding of the molecular mechanisms underlying PEDV pathogenesis and highlighting diltiazem as a potential antiviral candidate for PEDV infection.

Understanding how Cav1.2 affects PEDV infection is essential for the development of PED-targeted antiviral drugs. We found that Cav1.2 interacts with the S1 subunit of the PEDV S protein. A previous study reported that heparan sulfate (HS) acts as a cell attachment factor for PEDV infection [31]. Interestingly, Cav1.2 has also been reported to interact directly with HS via its first pore-forming domain [43]. It is possible that the trimetric S protein of PEDV, together with HS and Cav1.2, forms a much more stable complex that facilitates cell binding and infection. Our data show that Cav1.2 knockdown reduced PEDV binding; however, residual infection persists, suggesting the involvement of additional host receptors. A recent study found that Cav1.2 affects SARS-CoV-2 binding by regulating the cell surface expression of ACE2 [35]. Several host factors have been implicated in the PEDV entry process [23–32]; whether Cav1.2 affects PEDV binding by regulating the expression of these entry factors remains to be explored.

Diltiazem demonstrated anti-PEDV activity, with inhibition observed at both the binding and internalisation stages, suggesting that activated Cav1.2 is required for PEDV entry and that Ca^2+^-signalling may be involved in the process. A previous study found that Cav1.2 interacts with influenza virus hemagglutinin and mediates viral entry into host cells [34]. Influenza virus entry can induce a small, transient increase in Ca^2+^ concentration in areas where virus particles have been adsorbed prior to the broader increase in the entire cell Ca^2+^ level. Whether a similar local, Cav1.2-dependent Ca^2+^-signalling mechanism is involved in PEDV entry remains to be investigated. Similar roles for Cav1.2 in influenza and SARS-CoV-2 entry suggest a conserved calcium channel-dependent entry mechanism across diverse viruses, presenting a potential target for broad-spectrum antiviral strategies.

The time-of-addition assay showed that, compared with the viral RNA level of control cells, the addition of diltiazem at −1 h, 1 h, 2 h, or 4 h post-infection significantly decreased the viral RNA level, suggesting that diltiazem affects the early stages of PEDV infection. However, we cannot exclude the possibility that diltiazem may affect other stages of the viral life cycle. Viral internalisation assays further demonstrated that diltiazem significantly inhibited PEDV replication even after internalisation. Whether Cav1.2 influences stages beyond cell binding and internalisation remains to be studied. Given the continual emergence of PEDV recombinant mutations and new viral variants [44–47], it would be beneficial if diltiazem also targets other stages of PEDV infection, as this might reduce the risk of diltiazem-resistant PEDV strains developing.

While this study establishes the role of Cav1.2 in Vero-E6 cells, validation in porcine intestinal epithelial cells (the natural target of PEDV) is essential [48]. We will further explore Cav1.2’s role in the effects of PEDV infection in these cells in future work. Additionally, the precise structural interface between Cav1.2 and the PEDV S1 domain remains undefined. Cryo-EM or mutagenesis studies could help elucidate this interaction and guide the design of S protein inhibitors. Finally, in vivo trials are needed to assess diltiazem’s efficacy in pigs, balancing antiviral activity with potential off-target effects on calcium homeostasis.

In summary, we identify Cav1.2 as a critical entry receptor for PEDV, mediating viral binding and internalisation through interaction with the S1 subunit. The blockade of Cav1.2 by diltiazem represents a promising therapeutic strategy against PEDV. These findings advance our understanding of PEDV pathogenesis and underscore the broader role of calcium channels in viral entry, offering a potential antiviral target for combating emerging coronavirus-related diseases.

## Data Availability

All data generated or analysed during this study are included in this published article.
